# New insights of polyamine metabolism in testicular physiology: A role of ornithine decarboxylase antizyme inhibitor 2 (AZIN2) in the modulation of testosterone levels and sperm motility

**DOI:** 10.1371/journal.pone.0209202

**Published:** 2018-12-19

**Authors:** Ana Lambertos, Bruno Ramos-Molina, Andrés J. López-Contreras, Asunción Cremades, Rafael Peñafiel

**Affiliations:** 1 Department of Biochemistry and Molecular Biology B and Immunology, Faculty of Medicine, University of Murcia, Murcia, Spain; 2 Instituto Murciano de Investigación Biosanitaria (IMIB), Murcia, Spain; 3 Department of Pharmacology, Faculty of Medicine, University of Murcia, Murcia, Spain; University of Hyderabad, INDIA

## Abstract

The specific role of polyamines in the testis physiology is not fully understood. Antizymes (OAZs) and antizyme inhibitors (AZINs) are modulators of ornithine decarboxylase (ODC), a key enzyme in polyamine biosynthesis and polyamine uptake. Although the three known OAZs are expressed in the testis, only OAZ3 is testis specific and has been proven to have an essential role in male fertility. Regarding the two existing AZINs, AZIN2 is the most abundantly expressed member in this gonad. Whereas previous studies suggested that AZIN2 might participate in mouse spermatogenesis, immunohistological analysis of human testicular sections revealed that AZIN2 is also detected in the steroidogenic Leydig cells but not in the germinal epithelium. In the present study, we found a close ontogenic similarity in the mRNA levels of OAZs and AZINs between mice and rats, but an opposite expression pattern of ODC activity. Further analysis of AZIN2 and OAZ3 in the testis of mice with different alterations in spermatogenesis and fertility, induced either genetically or pharmacologically, corroborated that both AZIN2 and OAZ3 are mainly expressed in the haploid germinal cells. Finally, by using transgenic mice with a truncated *Azin2* gene fused to the bacterial *lacZ* gene, we studied the expression of *Azin2* in testes, epididymides and spermatozoa. AZIN2 was detected in spermatids and spermatozoa, as well as in Leydig cells, and in epithelial epidydimal cells. *Azin2* knock-out male mice were fertile; however, they showed marked decreases in testicular putrescine and plasma and testicular testosterone levels, and a dramatic reduction in the sperm motility. These results suggest an important role for AZIN2 in testicular cells by modulating polyamine concentrations, testosterone synthesis and sperm function. Overall, our data corroborate the relevance of polyamine regulation in testis functions, where both AZIN2 and OAZ3 play fundamental roles.

## Introduction

The polyamines spermidine, spermine, and their precursor putrescine are small aliphatic polycations that participate in multiple cellular functions including cell growth, differentiation and apoptosis. They are present at millimolar levels in mammalian cells, mainly associated with nucleic acids and proteins, and affecting DNA and chromatin structure, transcription, translation, cell signaling and different ion channels [1–3]. Cellular levels of polyamines are tightly regulated through the modulation of their biosynthesis, catabolism, uptake and excretion. In the center of this control, there is an autoregulatory mechanism that affects both the activity of ornithine decarboxylase (ODC), the first key biosynthetic enzyme that produces putrescine, and the polyamine uptake [4, 5]. A main component of this regulatory circuit is the ODC antizyme (OAZ), a protein that is induced by increasing polyamine levels by a unique ribosomal frame-shifting mechanism [6–8]. OAZ binds and inactivates ODC, and stimulates ODC degradation by the 26S proteasome without ubiquitination [9]. OAZ also inhibits polyamine uptake by a completely unknown mechanism [10]. The second component is a protein named antizyme inhibitor (AZIN), which is highly homologous to ODC, but devoid of enzymatic activity. AZIN binds to OAZ and negates the inhibitory action of OAZ on ODC and polyamine transport [5, 11–13]. In mammals, the antizyme family is formed by at least three different members named OAZ1, OAZ2 and OAZ3 [14]. Furthermore, two antizyme inhibitors (AZIN1 and AZIN2) have been found in mammals. They share the capacity to interact with the three OAZs, but differ on tissue, cellular and subcellular location [15–17].

The specific role of polyamines in testis physiology is not fully understood [18]. This can be related with the complex cellular organization of this gonad, and the continuous wave of cell differentiation that takes place during spermatogenesis. In the testis, whereas the seminiferous epithelium, formed by germ cells and supporting Sertoli cells, is fundamental in the process of spermatogenesis, the Leydig interstitial cells participate in the production and secretion of testosterone. Most of the early studies on polyamines in the testis were focused on the analysis of ODC activity and polyamine levels in the testes of rats, and in different types of rat testicular cells [19–25]. Particularly interesting was the study analyzing the changes in ODC activity and ODC mRNA levels from birth to maturity in rat testes, showing that the rise in ODC mRNA levels was in marked contrast with the decrease in ODC activity [26]. In mice, the analysis of ODC mRNA expression during testicular development showed that its levels increased in pachytene spermatocytes and round spermatids [27, 28]. These studies suggested that polyamines might play an important role during late meiosis and early spermiogenesis. The generation of transgenic mice overexpressing ODC in the testis revealed that the transgenic male mice presented marked morphological and functional alterations in this gonad [29, 30]. Additional studies using this mouse model led to the suggestion that putrescine may have strict selective local stimulatory and inhibitory action on DNA synthesis during spermatogenesis, and that an excess amount of the diamine could be related with decreased fertility [31].

During this period, no studies on the possible implication of AZs or AZINs on the testis function were addressed. The simultaneous discovery of OAZ3 as a new member of the antizyme family, specifically expressed in the spermatids and spermatozoa of rodent and human testes [32, 33] led to the suggestion that this protein could participate in the control of ODC during spermiogenesis. The fact that OAZ3 knock-out male mice were found to be infertile, having normal testicular morphology but aberrant spermatozoa, reinforced the hypothesis that OAZ3 could play a key role in haploid germ cell differentiation, likely by regulating the local concentration of polyamines [34]. However, the finding that AZIN2, a new paralogue of ODC devoid of enzymatic activity [13], was expressed in the haploid germ cell of the mouse testis [35] added a new potential player to the regulatory circuit affecting polyamine levels. In fact, other studies had shown that AZIN2 was able to interact with the three AZs, and modulate ODC activity and polyamine uptake [13, 36, 37]. Furthermore, immunohistological studies on the expression of AZIN2 in human testes revealed that it is expressed in the steroidogenic Leydig cells but not in the germinal epithelium [38]. This discrepancy between the two species has not been solved yet. In order to carry out a more detailed analysis of AZIN2 expression in mouse tissues, we generated transgenic mice with a truncated *Azin2* gene, fused to the bacterial *lacZ* gene (coding for β-D-galactosidase) under control of the *Azin2* promoter. Our results showed that the recombinant protein was present not only in the testes and brain, but in other specialized cells of the pancreas and adrenal glands [39].

In the present work, we first made a comparison on the expression of ODC, OAZs and AZINs in the testes of mice and rats, during the first wave of spermatogenesis. Secondly, the levels of the mRNA of the mentioned genes in the testes of two knock-out (KO) mouse models associated to male infertility, or in mice treated with cyclophosphamide, were studied. Finally, we tested possible biochemical and functional changes in the testes of our *Azin2* KO mice in order to provide further information on the role of AZIN2 in the reproductive function in male mice.

## Materials and methods

### Animals

Swiss CD1 mice, Sprague–Dawley rats and transgenic *Azin2* knock-out mice in a C57BL/6 mixed background bred in the Service of Laboratory Animals of the University of Murcia were used. Animals (3–4 months old) were fed with standard chow and water *ad libitum* and maintained at 22° C and 55% relative humidity under a controlled 12-h light, 12 h dark cycle (light on from 0800 h), at the Service of Laboratory Animals (University of Murcia). All animal procedures were compliant with the international guidelines of animal welfare and approved by the Bioethics Committee (Comité Ético de Experimentación Animal; Universidad de Murcia; 26012011). Protocols were designed to minimize the number of experimental animals and their suffering. For the analysis of gene expression in the testes during the postnatal period, male Swiss CD1 mice and Sprague–Dawley rats of different ages were used. Transgenic *Azin2* knock-out mice had been generated as described previously [39]. Briefly, the mutant mice carried a β–Geo cassette inserted between exons 4 and 5 of the *Azin2* locus. Transgenic mice genotyping was carried out with Phire Animal Tissue Direct PCR kit (Thermo Scientific), according to manufacturer’s instructions, and using the following primers: *Azin2* forward (5’-GAGGAGTCACATCACCACACG-3´), *Azin2* reverse (5´-GCTTCATGGTA GACATATGC-3´) and V76 reverse primer (5´-CCAATAAACCCTCTTGCAGTTGC-´3). Animals were euthanized by cervical dislocation after light anesthesia with isoflurane, and tissues were dissected and used for different assays. Testes from ATM null mice and Seckel mice were kindly provided by Dr. Fernández-Capetillo (CNIO, Madrid).

### Treatments

Adult and 7-day-old CD1 mice or adult and 7-day-old Sprague-Dawley rats were treated with human chorionic gonadotropin (hCG) (Sigma) for 5h. Young animals received an intramuscular injection of 5 IU of hCG, whereas adult animals received 15 IU hCG. Experiments with cyclophosphamide (CP, Sigma) were carried out with adult CD1 male mice. Cyclophosphamide (200 mg/kg, dissolved in 0.1%NaCl) was administered once a week by intraperitoneal injection, during five weeks, and animals were sacrificed after periods of 1, 5 or 8 weeks of recovery. Control animals received injection of 0.1% NaCl.

### Ornithine decarboxylase activity analysis

Tissues were homogenized in 10 mM Tris-HCl pH = 7.2, 0.25 M sucrose, 0.1 mM pyridoxal phosphate, 0.2 mM EDTA and 1 mM dithiothreitol. The homogenates were centrifuged at 14,000 × g for 20 min, and the supernatants were used to measure enzymatic activity. ODC activity was determined by measuring ^14^CO2 released from 0.4 mM L-[1-^14^C] ornithine (specific activity 4.7 mCi/mmol). The reaction was performed in polypropylene tubes with tightly closed rubber stopper, hanging from the stoppers two disks of filter paper wetted in a solution of benzethonium hydroxide and methanol (1:1). The samples were incubated at 37°C from 15 to 120 min, and the reaction was stopped by adding 0.5 ml of 2 M citric acid. The filter paper disks were transferred to scintillation vials with 1.5 ml of scintillation liquid and counted in a Tri-Carb 2900TR scintillation counter (Perkin Elmer, USA).

### Analysis of β-D-galactosidase activity

For β-galactosidase analysis, tissues were homogenized by means of a Polytron homogenizer in 50 mM TRIS-HCl pH 7.4 containing 1 mM EDTA and 1% Igepal. Tissue homogenates were centrifuged at 12.000xg for 20 min. and β -galactosidase activity was assayed in the supernatants by measuring the rate of hydrolysis of the substrate o-nitrophenyl-β-D-galactoside (ONPG). The incubations were performed at 37°C for 30 or 60 min in buffers of various pHs (from pH = 2.7 to pH = 9.5), containing 2 mM MgCl_2_, 50 mM β-mercaptoethanol and 0.66 mg/ml of ONPG, in a total volume of 0.3 ml. The reaction was stopped by adding 0.7 ml of 500 mM sodium carbonate. After centrifugation at 12.000xg for 5 min the absorbance at 420 nm was measured and the activity was expressed as the increase in A_420_ per h and g of wet tissue.

### β-D-galactosidase reporter staining

Testes and epididymides were dissected and fixed in 4%PFA in PBS (pH 7.4) for 4 hours at 5°C. After fixation, tissues were transferred into 20% sucrose in PBS for 48 hours, embedded in OCT freezing medium, and snap-frozen in isopentane chilled on liquid nitrogen. Twelve μm thick sections were cut on a cryostat at -26°C and placed on poly-L-lysine coated slides. One mL of 1% X-gal (5-bromo-4-chloro-3-indolyl-galactopyranoside) in diethylformamide was added to the 50 ml of PBS (pH 7.4) containing 0.1 g MgCl_2_, 0.48 g potassium hexacyanoferrate(III) and 0.64 g potassium hexacyanoferrate(II) trihydrate. The frozen sections were incubated at 37°C in the X-gal solution in a humidity controlled incubator overnight, counterstained with Neutral Red, dehydrated and mounted with DPX medium. For X-Gal staining of spermatozoa, cauda epididymides from WT and *Azin2* KO mice were open in PBS media, and the released sperm cells were collected by centrifugation and incubated with X-gal solution until color development. Sperm cells were washed and resuspended in PBS 1X for picture capture.

### Combined histochemistry β-galactosidase detection and α-inhibin immunohistochemical determination

Testes from *Azin2* KO mice were slightly fixed in 2% neutral buffered formalin, pH 7.4, snap frozen and preserved at -80°C into OCT-compound medium until use. Five micrometers cryosections were then obtained from the samples and stored at -20°C until use. To determine the β-galactosidase activity in tissue, an X-gal histochemical determination was carried out. Briefly, sections were defrozen for 10 minutes, fixed in acetone for 20 minutes at room temperature (RT) and incubated with an X-gal solution under dark, in humidity chamber racks at 37°C for 5 hours. After the β-galactosidase determination, a standard ABC-immunohistochemical procedure was then carried out in the same sections. Therefore, endogenous peroxidase was blocked for 5 minutes at RT by using a commercial solution (Dako peroxidase blocking reagent, Agilent Technologies, Barcelona, Spain). Sections were then incubated for 20 minutes at 37°C in normal goat serum (Vector Laboratories, Burlingame, CA, USA) to prevent background, and with a prediluted monoclonal mouse anti-inhibin α (Dako) for 40 minutes at 37°C. After washing, sections were then incubated with a secondary goat-anti mouse labelled polymer (Dako Envision Flex, Dako) for 15 minutes at 37°C. Positive immunoreaction was finally revealed by incubation with 3,3`-diaminobencidine (DAB) commercial solution for 3 minutes to prevent background. Positive immunoreaction was identified as a dark-brown precipitate. Finally, sections were counterstained with a neutral red solution for 3 minutes at RT and mounted in a aqueous mounting medium (VectaMount, Vector).

### Polyamine content

Testes were homogenized in 0.4 M perchloric acid (1:10 w/v), and the supernatants obtained after centrifugation at 12,000xg for 10 min were used for polyamine analysis by HPLC. For this purpose 100 μl of supernatant were mixed with 200 μl of saturated sodium carbonate and 400 μl of dansyl chloride (10 mg/ml in acetone) and incubated at room temperature overnight. Dansylated polyamines were extracted with toluene and separated by HPLC using a Bondapak C18 column (4.6 × 300 mm) and acetonitrile/water mixtures (running from 70:30 to 96:4 during 30 min of analysis) as mobile phase and at a flow rate of 1 ml/min. 1,6-Hexanediamine and 1,7-diaminoheptane were used as internal standards. Detection of the derivatives was achieved using a Waters 420-AC fluorescence detector, with a 340-nm excitation filter and a 435-nm emission filter.

### Testosterone analysis

Blood from KO and wild type mice was collected by cardiac puncture under isoflurane anesthesia and plasma was isolated by centrifugation. For the analysis of testicular testosterone, testes from KO and wild type mice were homogenized in 100% ethanol (1:20 w/v) and the supernatant obtained after centrifugation at 10000xrpm for 20 min and was diluted conveniently with 50% ethanol in physiological saline solution. Testosterone was measured using a Testosterone ELISA kit (IBL), according to the manufacturer’s instructions.

### Sperm quality assay

The analysis of WT and *Azin2* KO sperm quality was carried out by ETYCA Research (Barcelona) where sperm concentration, motility and morphology were assessed.

### Quantitative real-time RT-PCR

Testes were homogenized in Trizol and total RNA was extracted with Pure Link RNA Mini Kit (Life Technologies) following the manufacturer’s instructions. First-strand cDNA was obtained from total RNA using MMLV reverse transcriptase. One to 5 μg of total RNA was reverse-transcribed using 1 μl oligo (dT) as primer, 1 μl of 10 mM dNTP Mix, 1 μl of MMLV reverse transcriptase, 2 μl of buffer (containing 500 mM Tris-ClH pH 8.3, 50 mM KCl, 3 mM MgCl_2_ and 5 mM DTT) and nuclease-free water up to 20 μl. RNA, oligo dT and dNTPs were mixed and incubated at 70° C for 10 min. Mixture was put on ice for a few minutes and MMLV reverse transcriptase was added. After incubation at 37°C for 1 h, the transcriptase was denatured by heating at 90° C for 10 min. PCR amplification was carried out using a SYBR Green PCR Master Mix (Applied Biosystems) and a 7500 Real-Time instrument (Applied Biosystems). Different sets of primers (S1 Table) and cDNA were used, and the fluorescence data were collected and analyzed using 7500 SDS software (Applied Biosystems). The expression level of each gene was normalized against beta-actin.

### Microarray expression study

RNA extraction was performed according to the procedure previously described. The quality of RNA samples was determined by a capillary electrophoresis system (2100 Bioanalyzer, Agilent Technologies). Next, cDNA was synthesized, amplified and fragmented for hybridization with Mouse Gene 1-1-ST Array (901628 Affymetrix) using a Gene Atlas Microarray System (Affymetrix). Microarray data analysis was carried out by using the following software: Gene Atlas Instrument Control and Transcriptome Analysis Control. The raw data are available from Gene Expression Omnibus (GEO), accession number GSE121518.

### Statistical analysis

Statistical significance was determined by one-way analysis of variance (ANOVA) followed by Tukey’s multiple comparison test, or by the Student’s t-test using the GraphPad Prism software. For linear correlation analyses, the Pearson correlation coefficient was calculated by using the GraphPad Prism software. Differences with a P-value <0.05 were considered significant.

## Results

### Developmental changes in the expression of ODC, OAZs and AZINs in the testes of mice and rats

**Fig 1:**
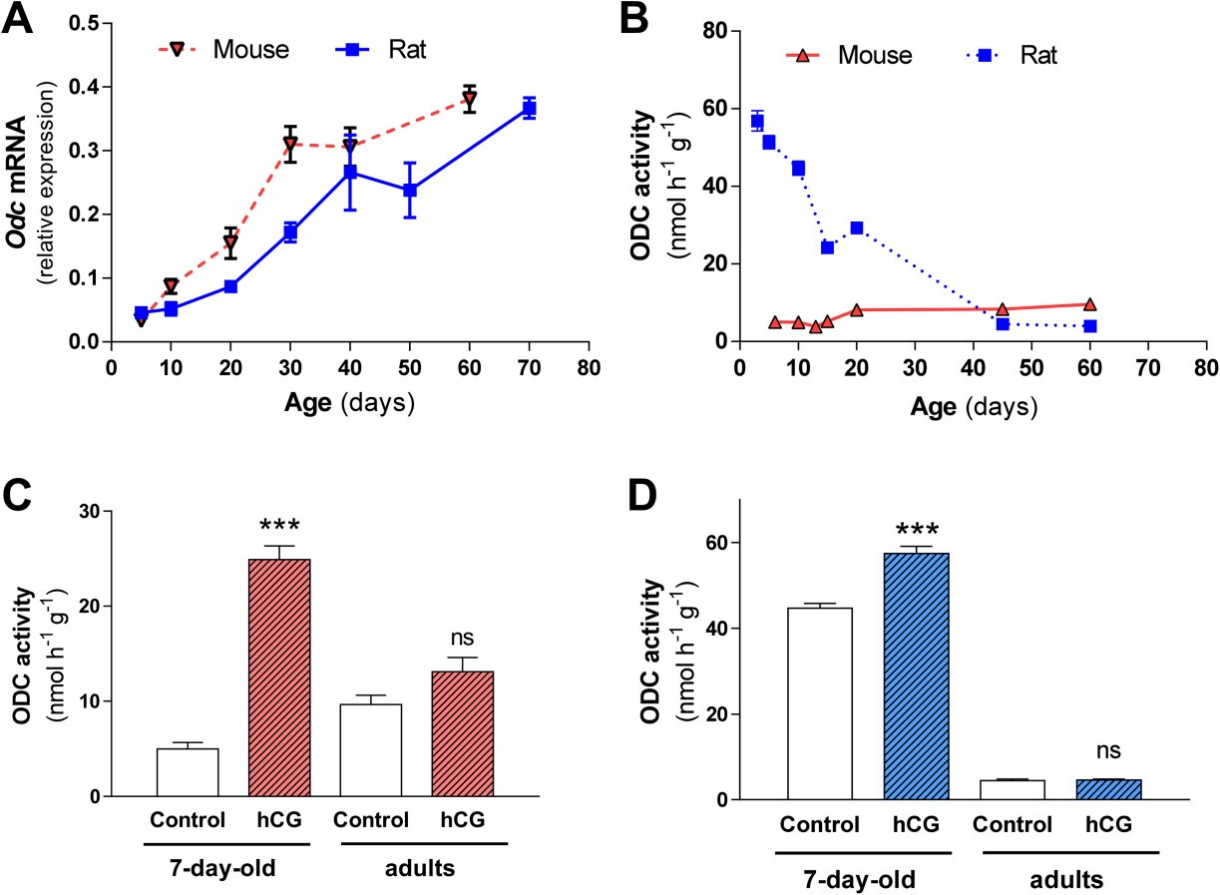
Comparative expression of ODC mRNA levels and ODC activity in testes of mice and rats during the postnatal period. **Effect of treatment with human chorionic gonadotropin (hCG).** A) RNA from a testis of CD1 mice and Sprague-Dawley rats at different ages was isolated and analyzed by real time RT-PCR. Expression values were normalized with respect to β-actin (Pearson correlation coefficient r = 0.9483, P = 0.0034 for mice; r = 0.9754, P = 0.0002 for rats). B) ODC activity was assayed in the contralateral testis of mice and rats at different ages (Pearson correlation coefficient r = 0.8727, P = 0.014 for mice; r = -0.9287, P = 0.0025 for rats). C) ODC activity in testes of young and adult mice after the treatment with hCG for 5h D) and rats (blue) ODC activity in testes of young and adult rats after the treatment with hGG for 5h. Results are expressed as mean ± SEM of 3 animals per group (A,B) or 5 animals per group (C,D). Statistical significance: *** P<0.001 *vs* its control group; ns, not significant.

We first compared the changes in ODC activity and ODC mRNA levels in the testes of mice and rats during the postnatal period. The relative levels of ODC mRNA steadily increased with age, with a rise of about ten-fold from post-natal day 5 to the adult age, in both mouse and rat testis (Pearson correlation coefficient r = 0.9483, P = 0.0034 for mice; r = 0.9754, P = 0.0002 for rats) (Fig 1A). However, the changes of testicular ODC activity along the first weeks of life were markedly different between species (Fig 1B). Whereas in mice the testicular ODC activity progressively increased with age (r = 0.872, P = 0.010), reaching at the day 60 values about 90% higher than at day 5, in the rats there was a marked decrease during the same period (r = -0.9287, P = 0.0025), with values in the adult testes about 7% of those of 3-day-old rats. In addition, during the first two weeks, the testicular ODC specific activity was markedly higher in the rats. This interspecies difference was also manifested after hormonal treatment of neonatal animals with human chorionic gonadotropin (hCG). In the testis of 7-day-old mice the acute treatment with the hormone produced a marked and significant increase in ODC activity (≈400%, P<0.001) compared to that found in rats (≈28%, P<0.001). The testes of adult animals were almost unresponsive to the hCG treatment (Fig 1C and 1D).

**Fig 2:**
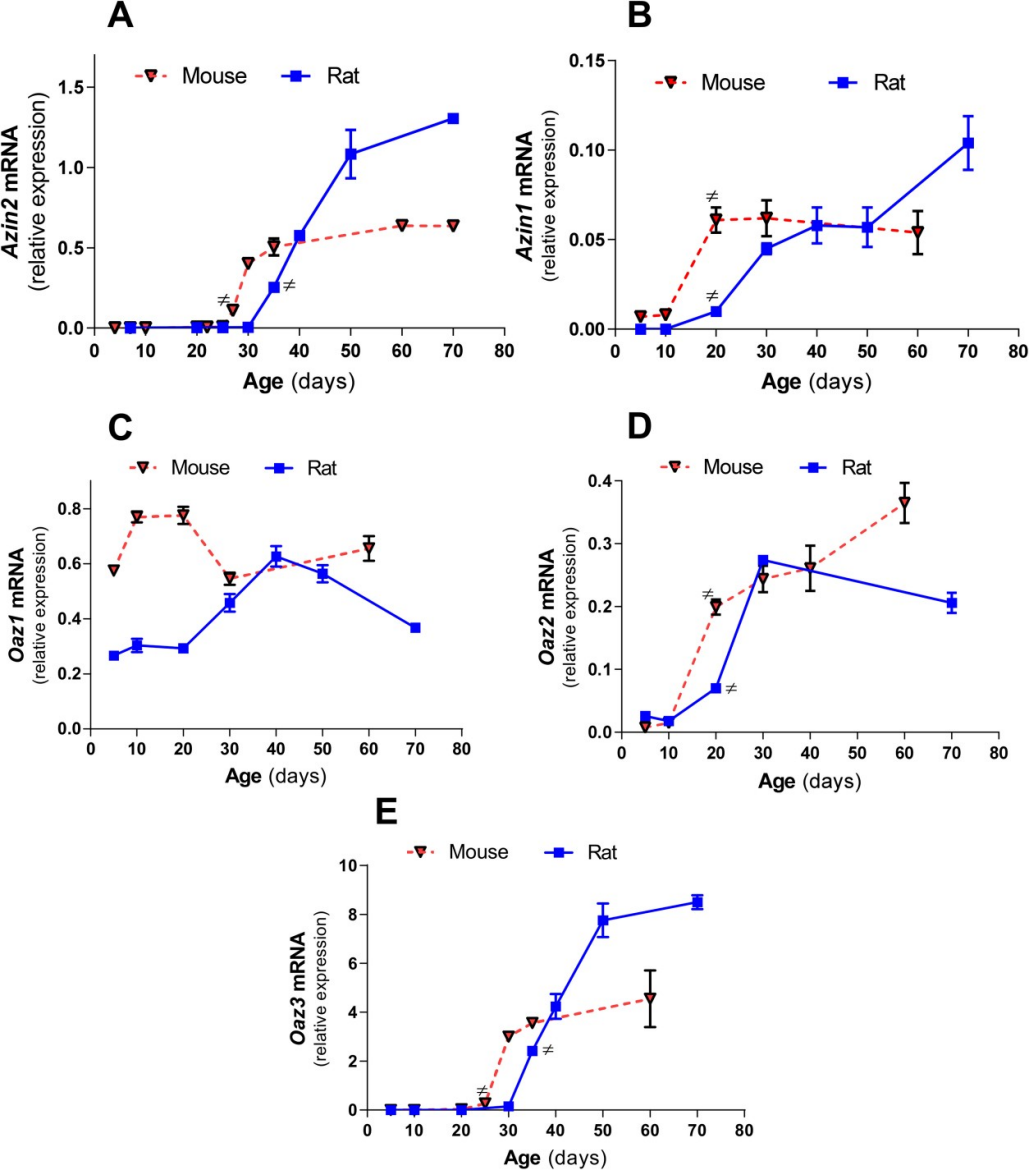
Comparative expression of ODC mRNA levels of *Azin*s and *Oaz*s in the testes of mice and rats during the postnatal period. mRNA levels were determined by real time RT-PCR. Expression values were normalized with respect to β-actin. Data represent mean±SEM of 2–3 animals per group until day 10, and 3–4 animals per group thereafter. Note that for all genes examined, except for *Oaz1*, the maximal increases in gene expression in mice took place before those of rats. (#) First value in the time series that is significantly different (P<0.01) from its precedent value.

The expression levels of OAZs and AZINs in the testes during the first wave of spermatogenesis were also analyzed by quantitative RT-PCR (Fig 2). In the mouse, the expression of AZIN2 and OAZ3 mRNA was non-detectable until the 4^th^ week of life whereas in the rat the expression of both genes began at the 5^th^ week (Fig 2A and 2E). In both cases, there was a marked temporal parallelism in the expression of both genes. This onset of AZIN2 and OAZ3 expression corresponds with the appearance of the round spermatids in the testis. Fig 2A and 2E also show that the transcription of these genes increased throughout the period in which differentiation from spermatids to spermatozoa takes place. The delay observed in rats compared with mice is likely related with the longer duration of the spermatogenic cycle in the rat. Similarly to AZIN2 and OAZ3, the expression profile of AZIN1 and OAZ2 increased during the post-natal period, but showed an earlier onset (Fig 2B and 2D). On the contrary, OAZ1 was already expressed in the testes of mice and rats during the first week of life, showing small changes during the analyzed period (Fig 2C). These results suggest that *Azin2* and *Oaz3* are mainly expressed in the testis during spermiogenesis.

### Changes in AZIN2 expression in the testes of adult mice treated with cyclophosphamide

**Fig 3:**
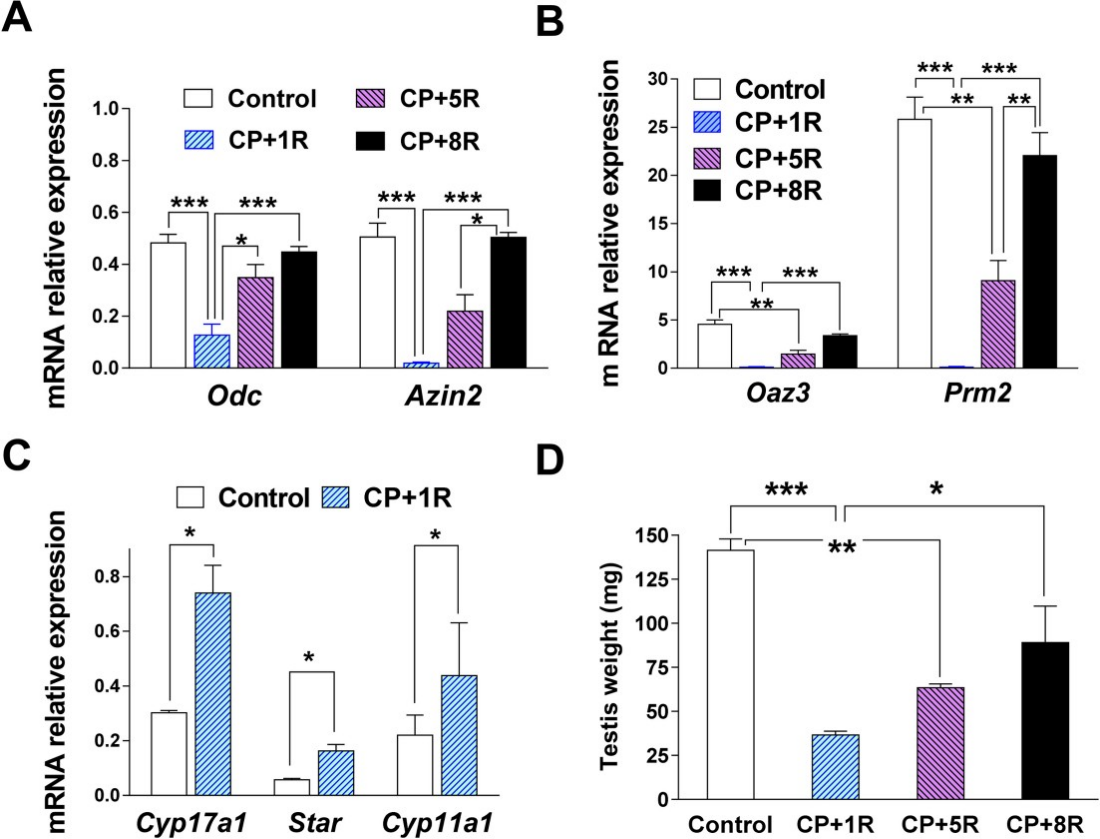
Effect of cyclophosphamide on testis mRNA levels and testis weight after the 1st, 5th and 8th weeks of last injection. Adult CD1 male mice received 200 mg/kg cyclophosphamide (CP) administered once a week, during five weeks, and animals were sacrificed after periods of 1 (CP+1R), 5 (CP+5R) or 8 (CP+8R) weeks of recovery (R). Expression values were normalized with respect to β-actin. *Odc* and *Azin2* (A); *Oaz3* and *Prm2* (B); steroidogenic genes *Cyp17a1*, *Star*, and *Cyp11a1* (C); Testis weight (D). Statistical significance was determined by one-way analysis of variance (ANOVA) followed by Tukey’s multiple comparison test. Data are shown as mean±SEM of 4 animals per group. * P<0.05, ** P<0.01, *** P<0.001.

Cyclophosphamide is a therapeutic anticancer agent that may cause testicular toxicity [40, 41]. In fact, it has been reported that treatment of mice with this drug produced severe damage of the seminiferous tubules and a marked decline in sperm production, aspects that were recovered after 5 weeks of treatment ending [42]. According to this report and to the data shown in Fig 2, the dramatic decrease in the formation of haploid testicular cells elicited by this drug should be associated with a marked fall in the levels of *Azin2* mRNA. To test this hypothesis, we treated adult mice with cyclophosphamide (200 mg/kg) for 5 weeks and analyzed the levels of *Azin2* mRNA and other mRNAs in the testis at the end of the treatment and after 5 or 8 weeks of recovery. As shown by Fig 3A and 3B, the levels of *Azin2* mRNA and *Oaz3* mRNA dramatically decreased (more than 90%) at the end of the treatment with cyclophosphamide, but they were completely recovered 8 weeks after ending the treatment. These changes were identical to those measured for protamine 2 (*Prm2*) (Fig 2B), a gene that is exclusively expressed in the haploid phase of the spermatogenesis. Contrary to the fall in these mRNA, those corresponding to the steroidogenic genes *Cyp17a1*, *Star* and *Cyp11a1*, which are expressed mainly in the Leydig cells not only did not diminish by the treatment, but showed a significantly relative increase (Fig 3C). The testis weight also decreased, and it was not completely recovered even 8 weeks after the end of the treatment (Fig 3D).

### Expression of AZIN2 in the testes of transgenic mice with altered testicular functions

**Fig 4:**
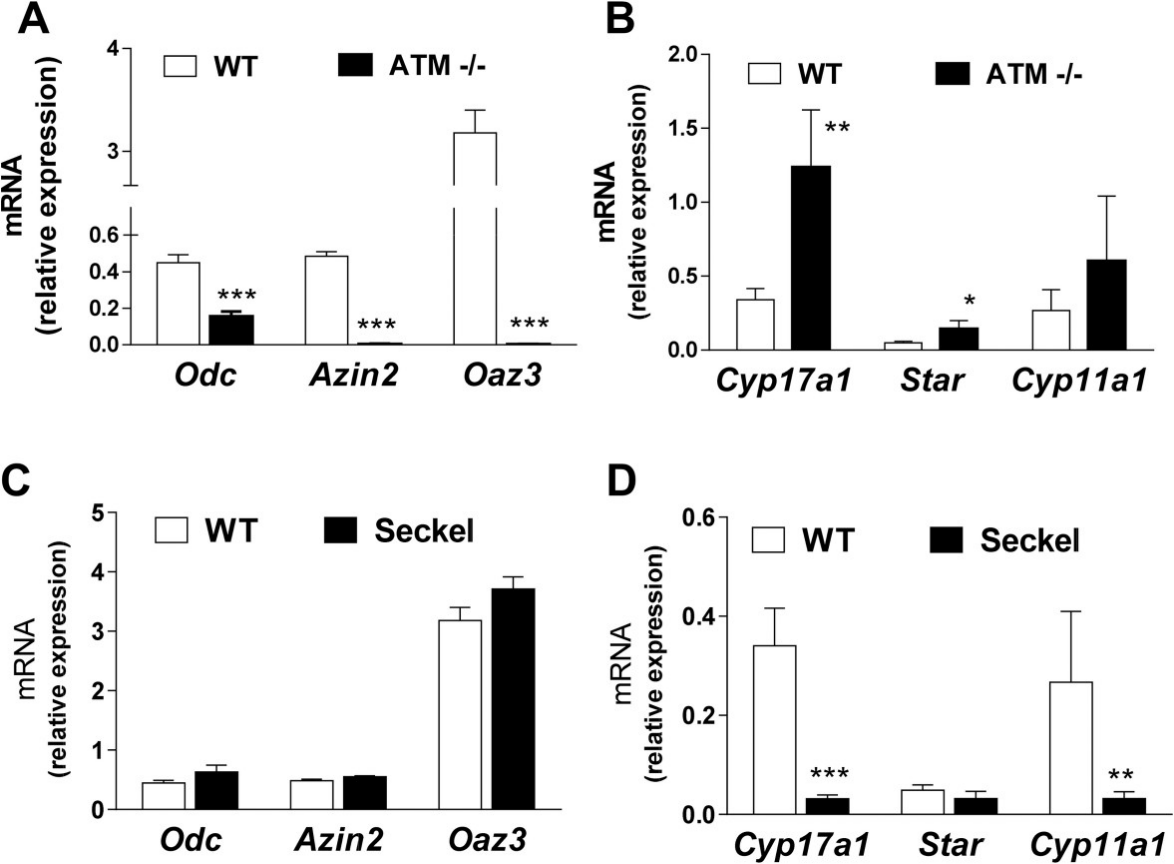
Comparative expression of polyamine related and steroidogenic related genes in testes of wild type mice and infertile models of mice. *Odc*, *Azin2*, and *Oaz3* mRNA levels were assayed by real time RT-PCR in *Atm*-null mice (A) and *Atr* hypomorphic (Seckel) mice (C). Steroidogenic enzymes mRNA levels were also assayed in in *Atm*-null mice (B) and *Atr* hypomorphic mice (Seckel) (D). Expression values were normalized with respect to β-actin. Statistical significance was determined by the Student’s t-test. Data are shown as mean±SEM of 3 animals per group. * P<0.05, ** P<0.01, *** P<0.001 *vs* their respective wild type mice.

In order to obtain further information on the types of testicular cells expressing *Azin2*, we analyzed the expression of *Azin2*, *Oaz*3 and *Odc* in the testis of two transgenic mouse models presenting male infertility: *Atm*-null mice and *Atr*-hypomorphic mice. Adult *Atm*-disrupted mice (a mouse model of Ataxia telangiectasia) have seminiferous tubules devoid of spermatids and spermatozoa, but with Leydig and supporting cells normal in appearance [43]. Mice with severe deficiency in ATR (a mouse model of Seckel syndrome) have small testes, and although infertile, successful in vitro fertilization could be achieved with ATR^S/S^ sperm [44]. The testicular levels of the different mRNA analyzed are shown in Fig 4. In the testis of *Atm*-null mice the expression of *Azin2* and *Oaz3* was almost non-existent (Fig 4A), in agreement with a lack of haploid cells. The expression of *Odc* was also reduced. On the other hand, the mRNA levels of some steroidogenic genes such as *Cyp17a1* and *Star* showed a relative increase (Fig 4B), as found earlier in the cyclophosphamide treated mice. In the case of the testes from Seckel mice, no changes in the expression of *Odc*, *Azin2* and *Oaz3* were detected (Fig 4C). Interestingly, a marked and significant decrease in the expression of *Cyp17a1* and *Cyp11a1* in the testis of Seckel mice was evident (Fig 4D).

### Analysis of the expression of AZIN2 and OAZ3 in human testicular germ cell tumors

**Fig 5:**
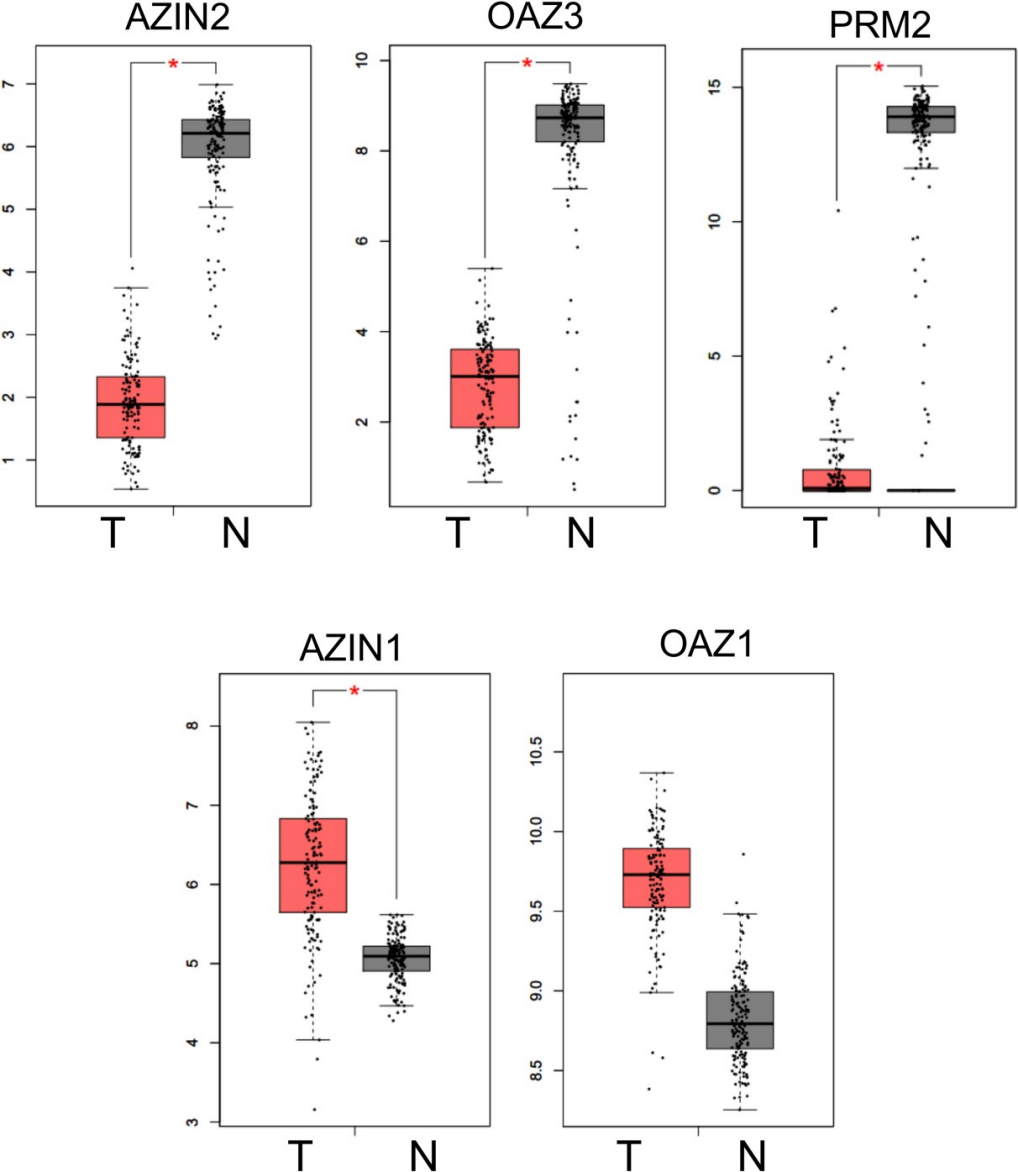
Boxplots comparing the expression of *AZIN2*, *OAZ3* and paralogues between human testicular germ cell tumors (T) and normal testes (N). Data were obtained from the analysis of current database using Gene Expression Profiling Interactive Analysis (GEPIA; http://gepia.cancer-pku.cn/) [45]. PRM2: protamine 2. Number of samples analyzed in each group: N = 165; T = 137. * P<0.05.

Since there are discrepant findings on the cellular location of AZIN2 in haploid cells between mouse and human testes [38], we decided to use the existing data on the gene expression profile between control and tumor testicular human cells to get complementary information on this subject. For that purpose, we used the GEPIA database (http://gepia.cancer-pku.cn/) [45] to compare the expression of *AZIN2* and *OAZ3* in human testicular germ cell tumors (N = 137) with that of normal tissue (N = 165). Fig 5 shows the Boxplots comparing the expression of *AZIN2*, *OAZ*3, *PRM2*, *OAZ1* and *AZIN1* in tumor and non-tumor testicular cells. Interestingly, a dramatic fall in *AZIN2* and *OAZ3* mRNA levels in the tumor cells was evident. The fact that this fall was comparable to that observed for *PRM*2, a gene expressed exclusively in the haploid germinal cells, suggest that *AZIN2* and *OAZ3* in the human testes might be mainly located in these testicular cells. The opposite was found for *AZIN1* and *OAZ1*, being this result consistent with a more general distribution of these proteins among the different testicular cells.

### Biochemical and histological study of the expression of *Azin2* in the testes of *Azin2* hypomorphic mice

**Fig 6:**
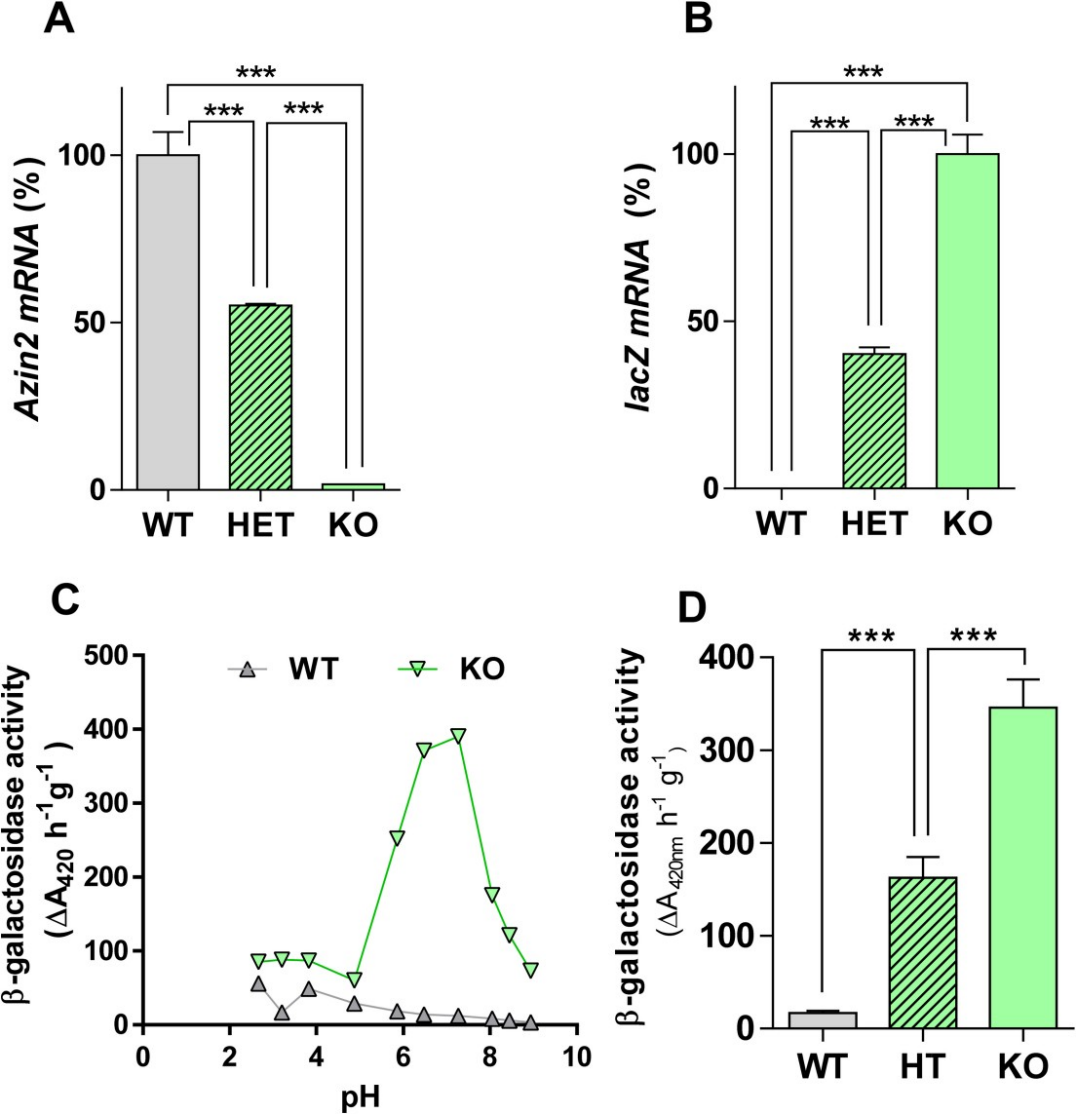
**Expression of *Azin2* (A), and *Azin2-lacZ* (B) in the testis of wild type (*Azin2*** ^**+/+**^**, WT), heterozygous (*Azin2*** ^**+/βGeo**^**, HET) or *Azin2* knock-out (*Azin2*** ^**βGeo/βGeo**^**, KO) adult mice. (C) Influence of pH on testicular β-D-galactosidase activity. (D) Neutral (pH = 7.2) β-D-galactosidase activity in the testis of WT, HET and KO adult mice.** For the analysis of *Azin2* mRNA levels a pair of primers amplifying exons 8 and 9 were used, and for lacZ the primers used amplified a segment of the bacterial enzyme. In A and B the values are expressed as mean±SEM of 3 animals per group. In C and D, β-D-galactosidase activity from testis homogenates is expressed as ΔA420 per h and g of wet tissue (means of triplicate determinations). Statistical significance was determined by one-way analysis of variance (ANOVA) followed by Tukey’s multiple comparison test. *** P<0.001.

**Fig 7:**
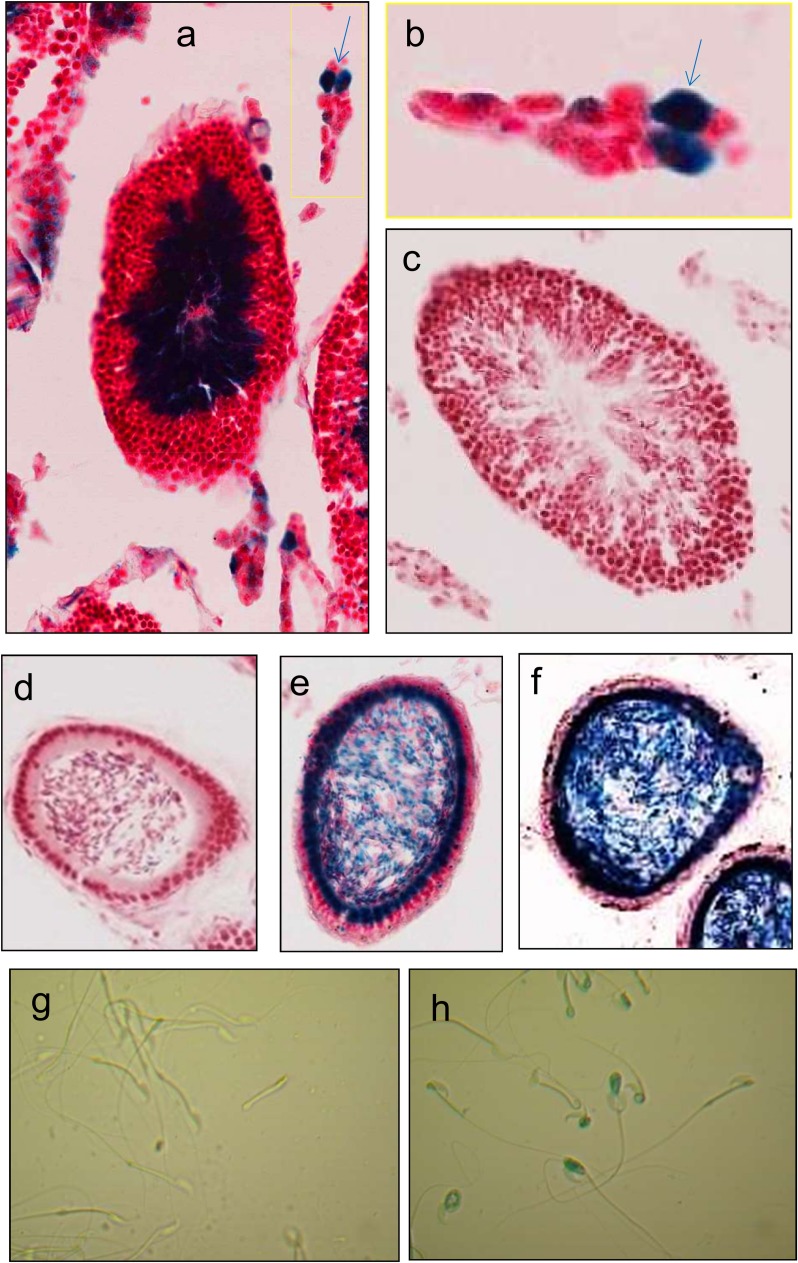
Histological X-Gal staining of the expression of *Azin2* in the testes, epididymides and sperm cells of WT and *Azin2* KO mice. a) Cross section of a seminiferous tubule from KO testis, showing positive X-gal staining (blue) in the inner part of the tubule where spermatids and spermatozoa are located (counterstained with neutral red). b) Magnification of interstitial cells; blue arrow indicates positive X-gal staining cells. c) Negative X-gal reaction in seminiferous tubule from WT testis. d) Cross section of epidydimis of WT mice. (e) Cross section of epidydimis of HET mice showing X-Gal stained (blue) and non-stained (red) spermatozoa. (f) Cross section of epidydimis of KO mice showing positive X-Gal staining of spermatozoa and epithelial cells. X-Gal staining of spermatozoa: negative in WT mice (g) and positive in KO mice (h).

In order to get a more precise information on the expression and function of AZIN2 in the mouse testis, we used a transgenic mouse model earlier generated in our laboratory [39]. The levels of *Azin2* mRNA were analyzed in the testes of homozygous *Azin2* hypomorphic mice (*Azin2* ^βGeo/βGeo^), as well as in heterozygous mice (*Azin2* ^+/βGeo^), and wild type mice (*Azin2* ^+/+^). The expression of *Azin2* in the homozygous mutant mice (KO) was about 1% of control values in WT, whereas in the heterozygous mice (HET) this value was about 50% (Fig 6A). As expected, the *lacZ* gene expression in the KO mice doubled that of HET mice and was non-detectable in the WT mice (Fig 6B). The β-galactosidase activity of testicular extracts was measured at different pHs. Fig 6C shows that in the testis of the KO mice a robust β-galactosidase activity was detected at neutral pH (consistent with the optimal pH of the bacterial enzyme encoded by *lacZ* gene), whereas in the WT mice such activity was almost nonexistent. In the HET mice this activity was about 50% from that of KO mice (Fig 6D). These results indicated that in the testis of KO mice, the recombinant protein, containing the N-terminal end of AZIN2 fused to the neutral bacterial β-galactosidase (NAZIN2-β-gal), was expressed under the control of the *Azin2* promoter, and therefore neutral β-galactosidase activity in the mouse transgenic tissues is a good reporter of the expression of wild type AZIN2 protein. Next, we examined the expression of neutral β-galactosidase in sections of testis and epididymis of KO mice, by means of histological X-gal staining, in order to detect the type of cells where AZIN2 protein is expressed. Fig 7A shows a cross-section of a seminiferous tubule showing intense blue staining (corresponding to β-galactosidase) in the inner part of the tubule, where spermatids and spermatozoa are located. No staining was observed in the seminiferous tubule of WT mice (Fig 7C). Interestingly, some blue staining was also detected in interstitial cells from KO testis (Fig 7B). In this case, X-Gal staining co-localized with cell immunostained with an antibody against Leydig cells (S1 Fig). The expression of *Azin2* was also examined in the mouse epididymis. Negative X-Gal staining was detected in cross-sections of control mouse epididymis (Fig 7D), whereas an intense staining was appreciated in both the epithelial cells and spermatozoa from HT and KO mice (Fig 7E and 7F). X-Gal staining of isolated spermatozoa obtained from the epididymides of WT and KO mice revealed positive staining in the case of KO mice (Fig 7G and 7H), corroborating that the recombinant protein NAZIN2-β-gal was still present in the spermatozoa.

### Influence of *Azin2* ablation on molecular and functional aspects of the testis

**Fig 8:**
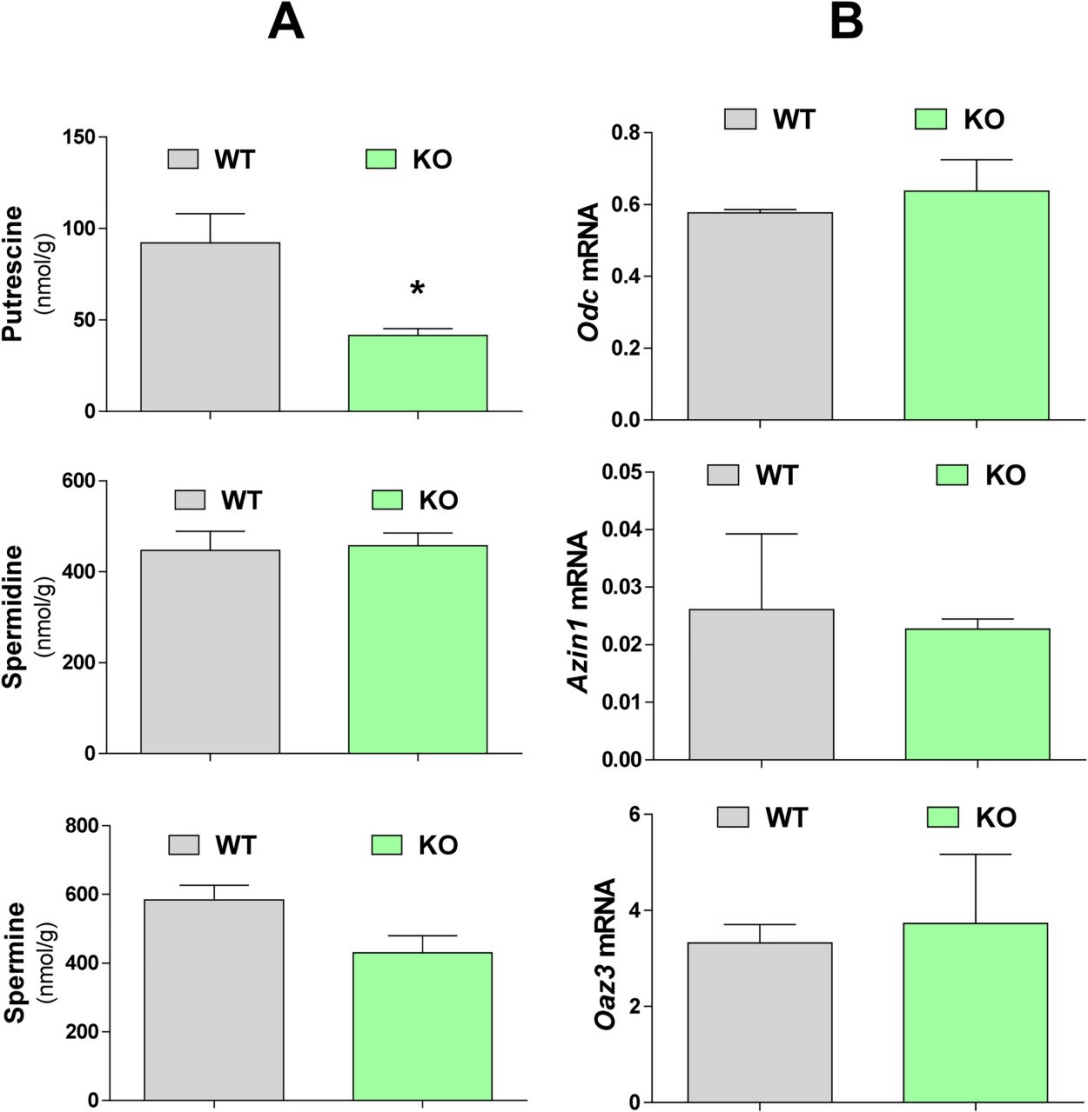
Comparison of polyamine levels and gene expression in testes of wild type (WT) and *Azin2* knock-out (KO) mice. (A) Polyamine levels were analyzed by HPLC (results are mean±SEM of 6 animals, 3 months old per group). (B) *Odc*, *Azin1* and *Oaz3* mRNA levels assayed by real-time RT-PCR analysis. Expression values were normalized with respect to β-actin (mean±SEM of 3 animals per group). Statistical significance was determined by the Student’s t-test. * P<0.05 *vs* WT.

**Fig 9:**
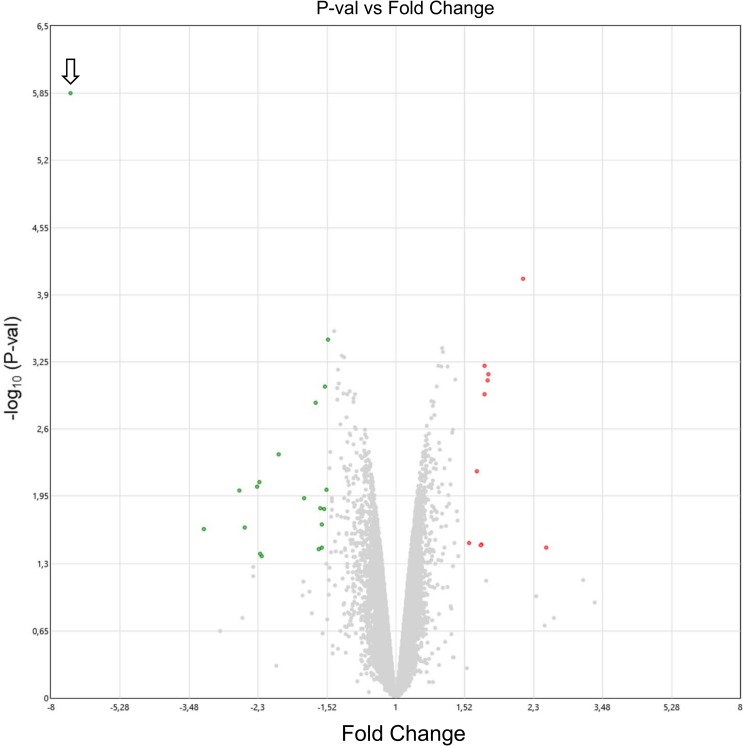
Volcano plot of microarray data. Dots represent the mean expression (n = 2) of individual genes obtained from a microarray normalized dataset. Cut-off values were established according to the following parameters: Fold change = ±1,5 and p-value <0,05. Genes above the cut-off standards are considered as differentially expressed genes and are highlighted as red and green dots, respectively. Genes with fold change above +1,5 are up-regulated in testes of *Azin2* KO mice, while genes with fold change below −1,5 are considered as down regulated in testes of *Azin2* KO mice. Note the prominent position of the *Azin2* gene (arrow), with a fold change value around -7,5. Each sample analyzed was a pool of RNA from three mice. The raw data are available from Gene Expression Omnibus (GEO), accession number GSE121518.

To analyze the possible molecular and functional consequences of the absence of AZIN2 in the testis, we compared some parameters between WT and *Azin2* KO mice. First, we examined the influence of AZIN2 in regulating total polyamine levels in the testes. The comparison of polyamine concentrations between the testes of WT and KO adult mice is depicted in Fig 8A. The levels of spermidine and spermine were unaltered. Interestingly, putrescine concentration in KO mice was reduced to approx. 40% of WT values (P<0.05). We analyzed, by quantitative RT-PCR, the mRNA levels of *Oaz3* and that of its paralogues *Odc* or *Azin*1 in the testis of KO mice, in order to know whether the suppression of *Azin2* may affect their expression. Fig 8B indicates that the lack of AZIN2 did not affect the mRNA levels of any of the three mentioned genes. Next, in order to detect how the disruption of *Azin2* could affect overall gene expression we performed microarray analysis of KO and WT mouse testes. Fig 9 shows a Volcano plot of microarray data. As expected, the gene mostly decreased in the KO mice was *Azin2* (fold-change -7,12; P<0.00001). Several genes were down-regulated or up-regulated (fold-change ±1.5; P<0.05). These genes are displayed on Table 1. None of them apparently has any relevant function related with testis physiology or polyamine metabolism.

**Table 1:** Several genes that are up-regulated or down-regulated (fold-change>±1.5, P<0.05) in the testes of *Azin2* KO mice compared to WT mice.

Fold Change	P-val	Gene Symbol	Description
-7,124	0,00001	Azin2	antizyme inhibitor 2
-2,572	0,00980	Vsig4	V-set and immunoglobulin domain containing 4
-2,486	0,02262	Fetub	fetuin beta
-2,310	0,00909	Zfp69	zinc finger protein 69
-2,276	0,00820	Slit2	slit homolog 2 (Drosophila)
-2,264	0,04072	Mirg; Mir410	miRNA containing gene; microRNA 410
-2,031	0,00440	Sult1e1	sulfotransferase family 1E, member 1
-1,739	0,01166	Sfi1; Drg1	Sfi1 homolog, spindle assembly associated (yeast); developmentally regulated GTP binding protein 1
-1,622	0,00138	Cxcl13	chemokine (C-X-C motif) ligand 13
-1,591	0,03667	Adh1	alcohol dehydrogenase 1 (class I)
-1,581	0,01456	Atp1a3	ATPase, Na+/K+ transporting, alpha 3 polypeptide
-1,567	0,03508	Gpha2	glycoprotein hormone alpha 2
-1,533	0,00098	Lilr4b	leukocyte immunoglobulin-like receptor, subfamily B, member 4B
-1,522	0,00966	Lrrn1	leucine rich repeat protein 1, neuronal
-1,506	0,00034	Ptpn14	protein tyrosine phosphatase, non-receptor type 14
1,556	0,03192	Cyp2c55	cytochrome P450, family 2, subfamily c, polypeptide 55
1,626	0,00646	Gm17535	predicted gene, 17535 [Source:MGI Symbol;Acc:MGI:4937169]
1,669	0,03356	Gm10324; Gm7762;	Human Ortholog ZNF700, zinc finger protein 700
1,678	0,03283	Gm10719; Gm10721	predicted gene 10719 [Source:MGI Symbol;Acc:MGI:3641690]; predicted gene 10721 [Source:MGI Symbol;Acc:MGI:3641688]
1,709	0,00061	Gprc5a	G protein-coupled receptor, family C, group 5, member A
1,742	0,00085	Gm24871; B930036N10Rik	predicted gene, 24871 [Source:MGI Symbol;Acc:MGI:5454648]; RIKEN cDNA B930036N10 gene [Source:MGI Symbol;Acc:MGI:3702496]
1,750	0,00074	Gm10718	predicted gene 10718 [Source:MGI Symbol;Acc:MGI:3642028]
2,154	0,00009	Cntfr	ciliary neurotrophic factor receptor
2,479	0,03507	9530002B09Rik	RIKEN cDNA 9530002B09 gene

**Fig 10:**
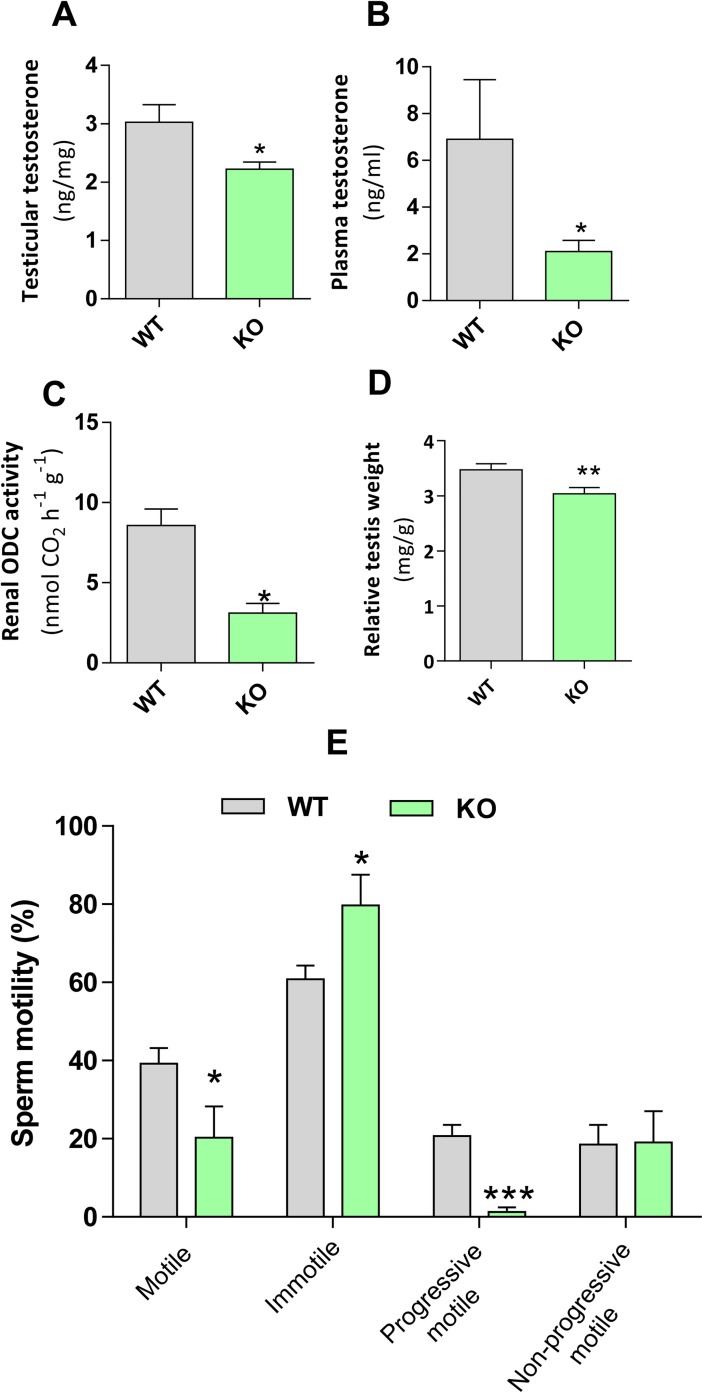
Influence of *Azin2* ablation on testosterone levels and sperm motility. Levels of testosterone in testes (A) and plasma (B) of WT and KO adult mice (n = 6 per group). C) Comparison of relative testis weight in adult WT and KO mice (n = 20 per group). D) Renal ODC activity of WT and KO male mice (n = 3 per group). E) Analysis of sperm motility expressed as percentage of cells from WT and KO mice (n = 4 per group) Statistical significance was determined by the Student’s t-test.*P<0.05, **P<0.01, ***P<0.001 *vs* WT.

To explore whether two important functions of the testis could be altered in the *Azin2* KO mice, we measured testosterone levels, sperm production and motility. As shown in Fig 10A and 10B, testosterone levels in both testes and blood plasma were markedly decreased in the KO mice (26% and 69%, respectively). In agreement with these findings, renal ODC activity, which is known to be stimulated by testosterone [46], decreased about 63% (Fig 10C). The analysis of the relative testicular weight revealed a small but significant reduction (13%; P<0.01) in the group of KO mice (Fig 10D). The analysis of sperm obtained from the cauda epididymides did not show significant morphological differences between KO and WT mice. Remarkably, significant changes were noticed in sperm motility (Fig 10E). *Azin2* KO mice had a lower percentage of motile sperm (20.2% vs 39%), although the major difference between WT and KO mice was observed in the progressive motility, with a much lower percentage of motile sperm in the KO mice (1.25%, P<0.001) compared to WT mice (20.7%). In spite of these differences, we did not detect reduced fertility in the KO male mice.

## Discussion

Although the metabolism and functions of polyamines in the reproductive system are only partially understood, there are many examples showing that both spontaneous and forced alterations of polyamines or proteins related with their metabolism, are associated with decreased fertility [18]. Regarding the role of polyamines on testis physiology, most studies have been conducted in rodents. Earlier experiments in rats were focused on analyzing ODC activity and its regulation by reproductive hormones [25–27, 47]. The development of the transgenic technology allowed to progress in our understanding on the role of polyamines on male mouse fertility [31, 34, 48, 49]. In the last years, the discovery of new potential regulators of polyamine metabolism such as AZIN2 or OAZ3, which are mostly or exclusively expressed in the mouse testis [32, 33, 35, 50], have provided new insights on the complexity of the polyamine network in this tissue. However, comparative studies on this subject between rats and mice are almost inexistent.

In the first part of the present work, we have carried out a comparative study on different aspects of polyamine biochemistry between mice and rats during the post-natal period. Our results indicate that there is a close parallelism between the evolution of *Odc* and *Azin1* mRNAs during this period. However, when the age-dependent variation of testicular ODC activity was analyzed, marked differences were observed, with a steady increase in mice and a marked fall in rats. This discrepancy was also manifested in the response to hormonal stimulation, since in young mouse testis ODC was highly responsive to hCG treatment, whereas in the rat of the same age the hormonal response was much lower. These results suggest that in the rat testis, during the early post-natal period, there is a translational or post-translational regulation of ODC different that does occur in mice. Although all OAZs and AZINs are known to be regulators of ODC activity in different experimental models [5, 14, 16], and therefore potential players in the testis function, little is known on the expression of the different members of these families in rodent testis. The present results clearly emphasize the relationship existing in the expression pattern of OAZs and AZINs in the testis of mice and rats. The fact that in the rat OAZ3 was not expressed during the first postnatal weeks, exclude the possibility that the lower rat testicular ODC above commented could be ascribed to this antizyme. However, the lower values in *Oaz1* expression in the rat testis during this period, may be responsible for the higher ODC activity. In addition, in the rat testis, the increases in the expression of OAZ3 and AZIN2 during the first wave of spermatogenesis are delayed with respect to mice, due to the longer duration of the rat spermatogenic cycle. This temporal pattern indicates that in rats, as in mice, the two genes could be expressed in spermatids and spermatozoa. Considering only the levels of mRNA, our results also show that OAZ3 is the antizyme most abundantly expressed in the rodent testis. Remarkably, whereas the expression of *Oaz1* was fairly constant in the mouse testis, in the rat there was a tendency to increase. The finding that the expression of *Oaz2* was also age-dependent and preceded that of *Oaz3*, suggests that *Oaz2* might be expressed in spermatocytes, without excluding its expression in spermatids. Taking into consideration these results, it can be postulated that each OAZ isoform could act preferentially in a specific type of testicular cell.

According to previous findings, there are some discrepancies between mice and human testes about the presence of AZIN2 in the haploid testicular cells [35, 38]. The results obtained in our present study in rats, support the contention that in this specie as in mice AZIN2 is also located in the haploid germinal cells. Furthermore, the parallel expression pattern of AZIN2 and OAZ3 with protamine 2 (PRM2, a specific marker of spermatids) in the testis of mice treated with cyclophosphamide, or in the two genetic murine models analyzed, provides stronger support to our initial claim [35] that AZIN2 is expressed in the haploid germinal cells. Consistently with this notion, the results seen in our *Azin2* transgenic mice clearly indicate that the AZIN2 protein is mainly present in this type of cells. In addition, the Gene Expression Profiling Interactive Analysis (GEPIA) of the database of human testicular tumors, showing a dramatic decrease in the expression of PRM2, AZIN2 and OAZ3 in the tumors versus normal tissue, profile completely different to that found for AZIN1 and OAZ1, indirectly suggests that AZIN2 is fundamentally expressed in human haploid testicular cells.

Our results with the *Azin2* transgenic mice, clearly showed that AZIN2 protein is mainly expressed in the inner part of the seminiferous tubules (Fig 7A), where spermatids at different stages of differentiation and spermatozoa are located. This finding is in full agreement with the results above commented based on mRNA analysis in mouse and rat testes (Figs 1–4). As expected, taking in consideration the positive action that AZIN2 exert on ODC activity, putrescine concentration significantly diminished in the testes of *Azin2* KO mice, with no relevant changes in spermidine and spermine. However, this does not exclude that in the testicular cells of the *Azin2* mutant, the subcellular location of polyamines may be different from that of WT testis, since it is known that AZIN2 is located in vesicular structures and it may affect the vesicular polyamine uptake [51,52]. In this context, male mutant mice with disruption of *Oaz3* were described as infertile, and their sperm presented alterations in the connection of the head with the tail, but surprisingly, the absence of OAZ3 did not affect polyamine concentrations in testes and epididymides [34]. Since *in vitro* studies have demonstrated that AZIN2 can interact with the three OAZs [13, 37], and in the testis AZIN2 and OAZ3 appear to share a regional and temporal expression, it seems likely that their co-expression should exert a fine regulation of ODC activity and polyamine uptake. However, several studies have raised some doubts about an exclusive role of OAZ3 on polyamine metabolism. On the one hand, it was found that OAZ3 was able to interact with gametogenetin, a protein expressed in spermatocytes and round spermatids [53]. On the other, it has been postulated that the major product encoded by *Oaz3* is a 12kDa protein that does not affect ODC levels, but interact with MYPT3, a protein regulator of the protein phosphatases PP1β and PP1γ2 [54]. Given the parallel expression of *Azin2* and *Oaz3*, it is plausible that AZIN2 may also regulate those polyamine-independent functions ascribed to OAZ3.

Our study with transgenic mice having *Azin2* ablation provided new information on the possible role of AZIN2 in the testis. It was evident that the gene was expressed not only in the spermatids but also in the testicular interstitial cells (likely Leydig cells), in the epididymis and in the epididymal spermatozoa, suggesting that AZIN2 could participate not only in the spermiogenesis but also in testosterone production. The marked decrease observed in the levels of testosterone in the testis and blood plasma of *Azin2* KO mice was consistent with the expression of this protein in the steroidogenic cells of the mouse testis shown here, and with that reported for human testes [38]. Notwithstanding the above, and due to the fact that *Azin2* is expressed in different brain regions, including the hypothalamus [55], it cannot be excluded that the transgenic mice may present some deficit in the hypothalamic-pituitary-gonadal axis that may also affect testosterone production. In addition to the reduced levels in testosterone, it was also patent that the lack of AZIN2 markedly decreased sperm motility. Although the male fertility was not significantly affected in our experimental settings, it is tempting to speculate that the higher motility observed in the sperm of WT mice as compared to *Azin2* KO mice has favored the expression of this protein in testis and its conservation during evolution. Taking into consideration all this evidence, and the commented specific subcellular location of AZIN2 [51, 52], it seems therefore likely that AZIN2, through its interaction with any of the three OAZs expressed in the testis, may participate in the regulation of local pools of polyamines during the process of spermiogenesis. However, the polyamine changes observed after suppression of AZIN2 appears to be insufficient to affect mouse fertility, in contrast to the lack of spermatozoa associated with marked increases in putrescine levels [29, 31] or decreases in spermine content [48]. Hence, further studies on the molecular mechanisms by which testicular polyamines may affect sperm properties are required.

Overall, this study provides new insights about the role of AZIN2 and polyamines in testis physiology. Our results not only indicate that there is a precise temporal and regional regulation of *Azin2* and *Oaz3* in rodent testis, but also that the deletion of *Azin2* results in reduced levels of putrescine and androgens, and disturbed sperm function. Together these findings suggest that alterations of the polyamine pathway may be related with certain human pathologies that affect testicular function.

## Supporting information

S1 Table (DOCX)

S1 Fig (TIF)
