# Supplementary material for: New insights of polyamine metabolism in testicular physiology: A role of ornithine decarboxylase antizyme inhibitor 2 (AZIN2) in the modulation of testosterone levels and sperm motility
Source: PLoS One. 2018 Dec 19;13(12):e0209202. doi: 10.1371/journal.pone.0209202 (PMC6300296; doi:10.1371/journal.pone.0209202)
Supplement: S1 Table — (DOCX) [file pone.0209202.s001.docx]

**Supplementary materials**

**S1 Table. Primers used in RT-PCR experiments.**

| **Gene** | **Forward (5’-3’)** | **Reverse (5’-3’)** |
| --- | --- | --- |
| *β-actin* (m) | GATTACTGCTCTGGCTCCTAGCA | GCTCAGGAGGAGCAATGATCTT |
| *β-actin* (r) | GATCACTGCCCTGGCACCCAGC | GATCACTGCCCTGGCACCCAGC |
| *Odc* (m,r) | ATGGGTTCCAGAGGCCAAA | CTGCTTCATGAGTTGCCACATT |
| *Oaz1* (m) | GAGTTCGCAGAGGAGCAACT | CCAAGAAAGCTGAAGGTTCG |
| *Oaz1* (r) | GAGTTCGCAGAGGAGCAGCT | CCAAGAAAGCTGAAGGTTCG |
| *Oaz2* (m,r) | AGTAAGTGTCCCCAGCTCCA | ATCTTCGACAGTGGGTGAGG |
| *Oaz3* (m) | CCAGGTGGGTAGGAGCACT | AAGCAGGGGGTCAGTTGATA |
| *Oaz3* (r) | CCAGGTGGGTAGGAGCACT | AAGCAGGGGGTCGGCTGATA |
| *Azin1* (m) | CTTTCCACGAACCATCTGCT | TTCCAGCATCTTGCATCTCA |
| *Azin1* (r) | CTTTGCACGGACCGTCTGCT | TTCCAGCATCTTGCATCTCA |
| *Azin2* (m,r) | GCTTAGAGGGAGCCAAAGTG | CTCAGCAAGGATGTCCACAC |
| *LacZ* | TTATCGATGAGCGTGGTGGTTATGC | GCACGATAGAGATTCGGGATTT |
| *Prm2* (m) | GGAGACACTCCTGCCGCCACA | CTGCACCTGCATCTCCTCC |
| *Cyp11a1* (m) | TCCTCAAAGCCAGCATCAAGG | CGAAGCACCAGGTCATTCAC |
| *Star* (m) | AAGGAAAGCCAGCAGGAGAAC | CCACATCTGGCACCATCTTACTTA |
| *Cyp17a1* (m) | ACAACTAGCTCTGTGCTGAACTGG | CACCTCAGGATTGTGCACCA |

(m) mouse; (r) rat; Odc, ornithine decarboxylase; Oaz, antizyme; Azin, antizyme inhibitor, LacZ, *E. coli* beta-galactosidase; Prm, protamine; Cyp, cytochrome P450; Star, steroidogenic acute regulatory protein

**S1 Fig. Combined histochemistry β-galactosidase detection and α-inhibin immunohistochemical determination in interstitial testicular cells from *Azin2* KO mice.** (A, B) Sections from mouse testis were first stained with X-Gal. After β-galactosidase determination the sections were analyzed by an ABC immunohistochemical procedure using an anti-inhibin α monoclonal antibody, as described in the Materials and Methods section. Note interstitial cells sharing both labels. (C) Section assayed only with the anti-inhibin α monoclonal antibody. Note that immunoreactivity was detected in Leydig cells but not in the cells of the seminiferous tubules.
