# Supplementary figures and images for: New insights of polyamine metabolism in testicular physiology: A role of ornithine decarboxylase antizyme inhibitor 2 (AZIN2) in the modulation of testosterone levels and sperm motility
Source: PLoS One. 2018 Dec 19;13(12):e0209202. doi: 10.1371/journal.pone.0209202 (PMC6300296; doi:10.1371/journal.pone.0209202)

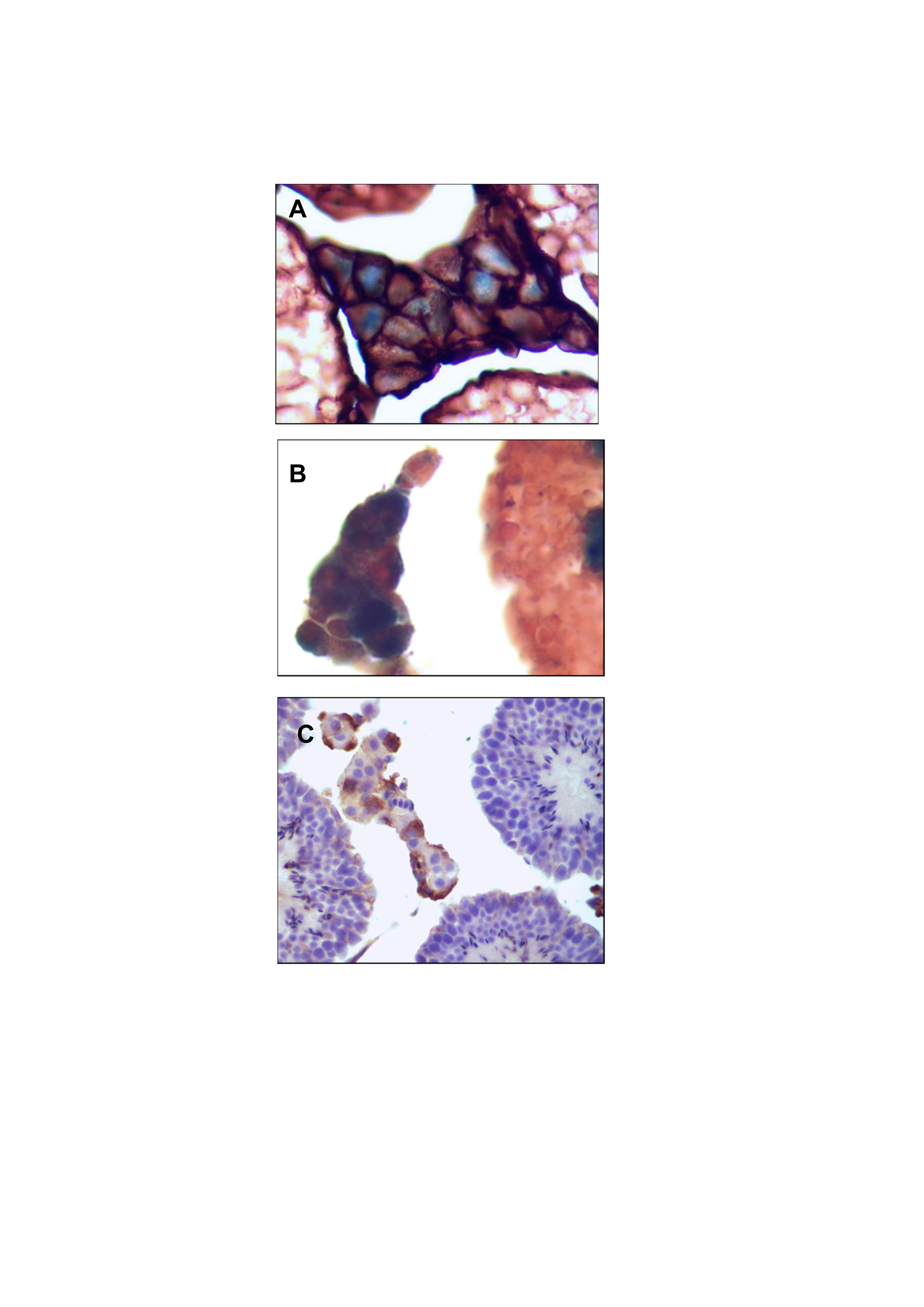

Supplement: S1 Fig — (TIF) [file pone.0209202.s002.tif]
